# Significant interarm blood pressure difference predicts cardiovascular risk in hypertensive patients: CoCoNet study

**DOI:** 10.1097/MD.0000000000003888

**Published:** 2016-06-17

**Authors:** Su-A Kim, Jang Young Kim, Jeong Bae Park

**Affiliations:** a Division of Cardiology, Department of Medicine, Cheil General Hospital, Dankook University College of Medicine, Seoul, Republic of Korea; b Division of Cardiology, Department of Internal Medicine, Yonsei University Wonju College of Medicine, Republic of Korea.

**Keywords:** hypertension, systolic interarm blood pressure difference, cardiovascular risk

## Abstract

There has been a rising interest in interarm blood pressure difference (IAD), due to its relationship with peripheral arterial disease and its possible relationship with cardiovascular disease. This study aimed to characterize hypertensive patients with a significant IAD in relation to cardiovascular risk. A total of 3699 patients (mean age, 61 ± 11 years) were prospectively enrolled in the study. Blood pressure (BP) was measured simultaneously in both arms 3 times using an automated cuff-oscillometric device. IAD was defined as the absolute difference in averaged BPs between the left and right arm, and an IAD ≥ 10 mm Hg was considered to be significant. The Framingham risk score was used to calculate the 10-year cardiovascular risk. The mean systolic IAD (sIAD) was 4.3 ± 4.1 mm Hg, and 285 (7.7%) patients showed significant sIAD. Patients with significant sIAD showed larger body mass index (*P* < 0.001), greater systolic BP (*P* = 0.050), more coronary artery disease (relative risk = 1.356, *P* = 0.034), and more cerebrovascular disease (relative risk = 1.521, *P* = 0.072). The mean 10-year cardiovascular risk was 9.3 ± 7.7%. By multiple regression, sIAD was significantly but weakly correlated with the 10-year cardiovascular risk (β = 0.135, *P* = 0.008). Patients with significant sIAD showed a higher prevalence of coronary artery disease, as well as an increase in 10-year cardiovascular risk. Therefore, accurate measurements of sIAD may serve as a simple and cost-effective tool for predicting cardiovascular risk in clinical settings.

## Abstract 2

Supplemental Digital Content is available in the text

## 1 Introduction

Since the early twentieth century, interarm blood pressure difference (IAD) has been recognized in clinical practice.^[1]^ Significantly large IAD has been considered a marker for the diagnosis of peripheral artery disease, and IAD has been expected to be related with cardiovascular risk. However, the clinical significance of IAD has not been elucidated. Furthermore, clinical guidance for the management of patients with a large IAD has not yet been established.

Previous studies have revealed an increase in the prevalence of large IAD in patients with hypertension^[2,3]^ and diabetes mellitus.^[4]^ A relationship between IAD and atherosclerotic diseases, including subclavian artery stenosis,^[5]^ coronary artery disease,^[6]^ and other peripheral artery disease,^[7,8]^ has also been found. Arteriosclerosis with increased arterial stiffness^[9]^ was investigated as a factor for increased IAD. However, most of these studies were performed in Western populations with small sample sizes involving specific disease groups. Furthermore, the methods defining IAD were different in each study. Considering that different methods result in different IADs,^[2]^ the comparison of IADs between groups, or the assessment of the prevalence of large IADs in specific conditions, is unavailable. To accurately evaluate IAD without overestimating the result, blood pressure (BP) should be measured simultaneously and repetitively in both arms.

The characterization of IAD as a prognostic factor that can independently predict overt cardiovascular disease may be helpful in the clinical setting, as it may prevent the need for invasive evaluations and reduce healthcare costs. Therefore, we prospectively established a large cohort consisting of hypertensive patients, evaluated IAD, and used the Framingham risk score to examine the relationship between IAD and cardiovascular risk.

## 2 Materials and methods

### 2.1 Study sample and data collection

The Cooperative Network Construction of a Nationwide Clinical Trial (CoCoNet) study is a prospective cohort study that is aimed to evaluate hypertensive patients’ characteristics and treatment strategies. Patients over 20 years old who were previously diagnosed with primary hypertension and received antihypertensive medication were included in the study. Patients who had systolic BP (SBP) ≥ 140 mm Hg or diastolic BP (DBP) ≥ 90 mm Hg and were newly diagnosed with primary hypertension were also included in the study. The exclusion criteria were as follows: a previous diagnosis of cardiac arrhythmia, systolic heart failure, or chronic kidney disease with hemodialysis, and patients who did not give informed consent. From September 1, 2013 to December 31, 2014, patients were enrolled from 10 primary clinics and 27 secondary and tertiary hospitals. All enrolled patients gave written informed consent, and study approval was obtained from the Institutional Review Board of Cheil General Hospital (IRB approval number: CGH-IRB-2013-33).

The patients’ body scale, medical history, and family history were collected. Routine blood chemistry tests, including lipid profiles and renal function tests, and electrocardiography were performed. For all coordinated laboratory results between hospitals, harmonization of the laboratory findings was completed.^[10]^ Diabetes mellitus was defined when patients were receiving oral hypoglycemic agents or insulin, or when patients had repeated results of fasting glucose ≥126 mg/dL or glycated hemoglobin ≥6.5%. Dyslipidemia was defined when patients were receiving lipid-lowering medications or when patients showed a fasting total cholesterol level of ≥220 mg/dL. Coronary artery disease and cerebrovascular disease were confirmed by chart review and history evaluations, which were assessed by a trained nurse. Symptoms presenting coronary artery disease were evaluated with electrocardiography and echocardiography with or without angiographic results by the judgment of the physician. Chronic kidney disease was confirmed when the estimated glomerular filtration rate was <60 mL/min.

### 2.2 BP measurements

Patients were asked to rest for at least 5 min, after which BPs were measured with an automatic cuff-oscillometric device (Watch BP office, Microlife, Taiwan). The device was set to measure BPs simultaneously 3 times in both arms at 2-min intervals. The BPs were then averaged to calculate the IAD. Systolic IAD (sIAD) was defined as the absolute difference in mean SBP between the left arm and right arm. Diastolic IAD (dIAD) was defined as the absolute difference in mean DBP between the left arm and right arm. Systolic and diastolic IADs ≥ 10 mm Hg were considered to be significant.

### 2.3 Ten-year cardiovascular risk evaluation

The Framingham risk score was used to estimate each patient's 10-year cardiovascular risk (%),^[11]^ based on age, smoking status, SBP, high-density lipoprotein cholesterol levels, and total cholesterol levels.^[12]^ Relationships between the 10-year cardiovascular risk and sIAD or dIAD were also evaluated.

### 2.4 Statistical analysis

Continuous variables were expressed as mean ± standard deviation. Bipartite variables were described as frequencies and percentages. The comparison of demographic parameters between patient groups divided by sIAD and dIAD was performed using the independent *t* test and chi-squared test. Differences in BP between the left and right arms were evaluated using the paired *t* test. The 3 BP measurements were compared using repeated measured ANOVA. Univariate and multivariate linear regression analyses were performed to evaluate the relationship between IAD and other parameters. Statistical significance was defined as a *P* value <0.05, and SAS version 9.3 was used for all statistical analyses.

## 3 Results

A total of 3699 hypertensive patients were enrolled (mean age, 61 ± 11 years, 52.8% male). The mean SBPs and DBPs were 128.2 ± 13.9 and 79.1 ± 9.9 mm Hg, respectively. SBPs and DBPs were significantly higher in male patients than in female patients, and pulse pressure was significantly higher in female patients. The prevalence of diabetes, dyslipidemia, coronary artery disease, cerebrovascular disease, or chronic kidney disease was 24.9%, 59.5%, 19.8%, 5.4%, or 4.8%, respectively, and more male patients had coronary artery disease and chronic kidney disease. At the time of enrollment, 94.6% of patients were taking antihypertensive medications (Table 1). The proportion of patients receiving combination therapy was 50.6%. There were small but significant differences in DBP between patients receiving monotherapy and combined therapy (SBP, 128.3 ± 14.2 mm Hg vs 127.7 ± 13.4 mm Hg, *P* = 0.160; DBP, 77.9 ± 9.8 mm Hg vs 79.7 ± 9.7 mm Hg, *P* < 0.001).

**Table 1:**
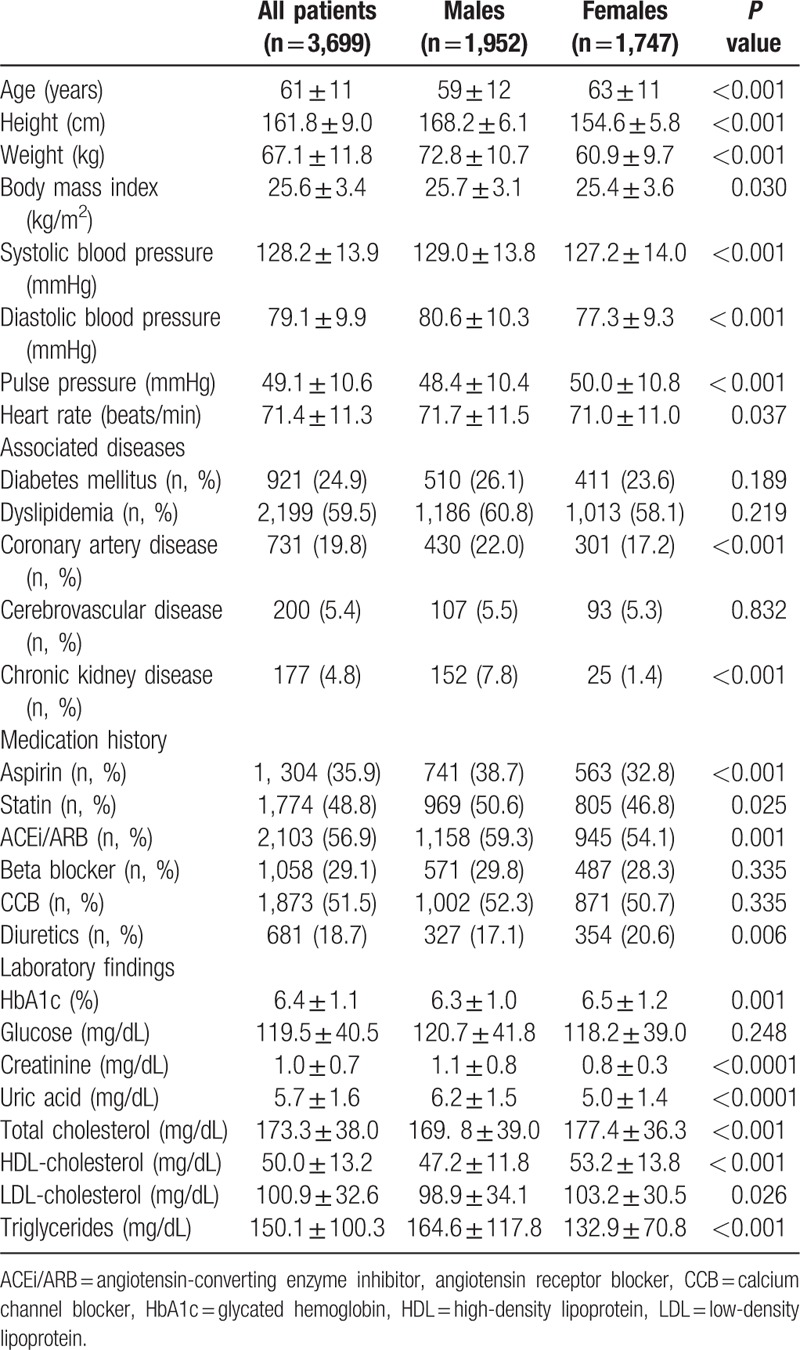
Baseline characteristics of all patients and comparison of baseline characteristics in male and female patients.

Table 2 shows the results of 3 repeated measurements of SBP and DBP, which were simultaneously measured in both arms. The 2nd and 3rd BP measurements were significantly lower than the 1st measurement, but there was no difference between the 2nd and 3rd measurements. The SBP of the left and right arms were significantly different in every measurement (*P* = 0.028, *P* = 0.027, *P* = 0.006, respectively), and the BP in the right arm was slightly higher than that in the left arm.

**Table 2:**
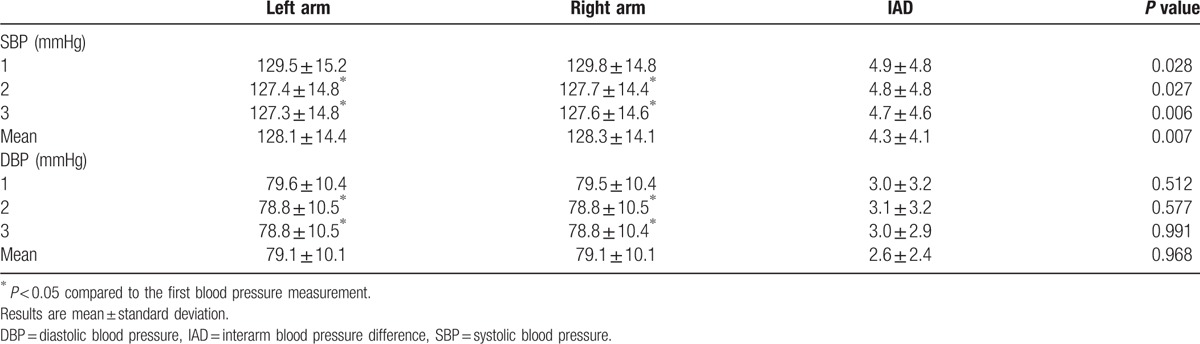
Both arm blood pressure measurements.

**Figure 1:**
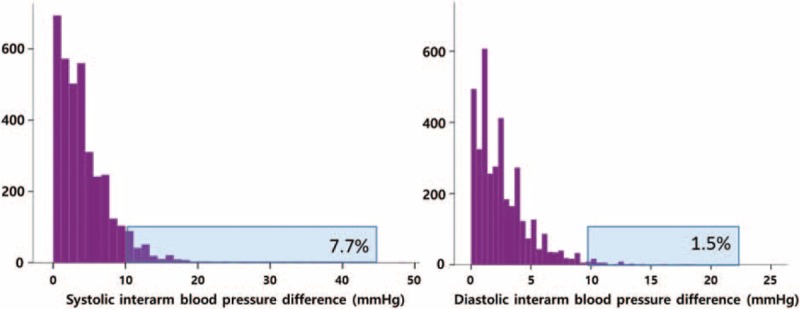
Distribution of systolic and diastolic interarm blood pressure differences.

The sIADs of the 1st, 2nd, and 3rd BP measurements were 4.9 ± 4.8 mm Hg, 4.8 ± 4.8 mm Hg, and 4.7 ± 4.6 mm Hg, respectively, and the sIAD of the mean BP was 4.3 ± 4.1 mm Hg. The dIADs of the 1st, 2nd, and 4rd measurements were 3.0 ± 3.2 mm Hg, 3.1 ± 3.2 mm Hg, and 3.0 ± 2.9 mm Hg, respectively, and the dIAD of the mean BP was 2.6 ± 2.4 mm Hg. The distribution of sIAD and dIAD by mean BP is shown in Fig. 1.

By simple regression analysis, the sIAD was not different by sex and did not correlate with age (Table 3). Multiple regression analysis results show that SBP (β = 0.016, *P* = 0.041) and body mass index (BMI) (β = 0.123, *P* < 0.001) were weakly but significantly associated with sIAD. The dIAD was weakly correlated with age, BMI, total cholesterol level, and SBP before and after multiple regression analysis (Table 3). In addition, sIAD was positively and significantly correlated with dIAD (r = 0.289, *P* < 0.001).

**Table 3:**
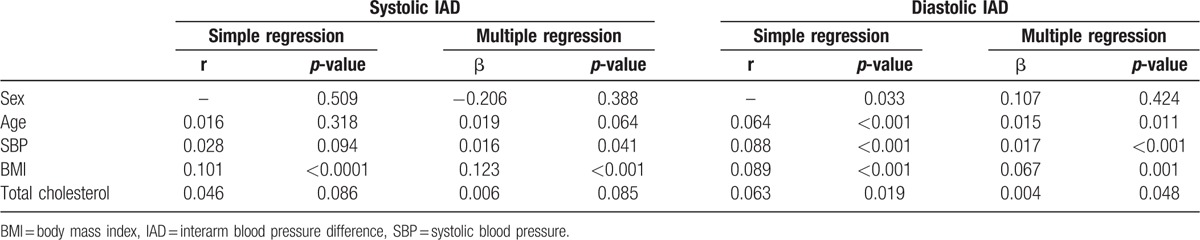
Simple and multiple regression analyses of systolic interarm blood pressure difference and diastolic interarm blood pressure difference with demographic parameters.

Two-hundred eighty-five patients (7.7%) showed significant sIAD (≥10 mm Hg), and 57 patients (1.5%) showed significant dIAD (≥10 mm Hg). Twenty-one patients (0.6%) showed both significant sIAD and dIAD. Patients with significant sIAD had higher weight, BMI, and pulse pressure than patients without significant sIAD (Tables 4 and 5). The SBP was also slightly higher in patients with significant sIAD than in patients without significant sIAD. Coronary artery disease and cerebrovascular disease were more common in patients with significant sIAD, with relative risks of 1.356 (*P* = 0.034, 95% confidence interval [CI] 1.022–1.800) and 1.521 (*P* = 0.072, 95% CI 0.960–2.410), respectively. These patients were more likely to receive beta blockers. Furthermore, patients with significant dIAD were older and had higher BMI, SBP, and pulse pressure than those without significant dIAD. There was no significant difference in the prevalence of cardiovascular disease and cerebrovascular disease between patients with and without significant dIAD.

**Table 4:**
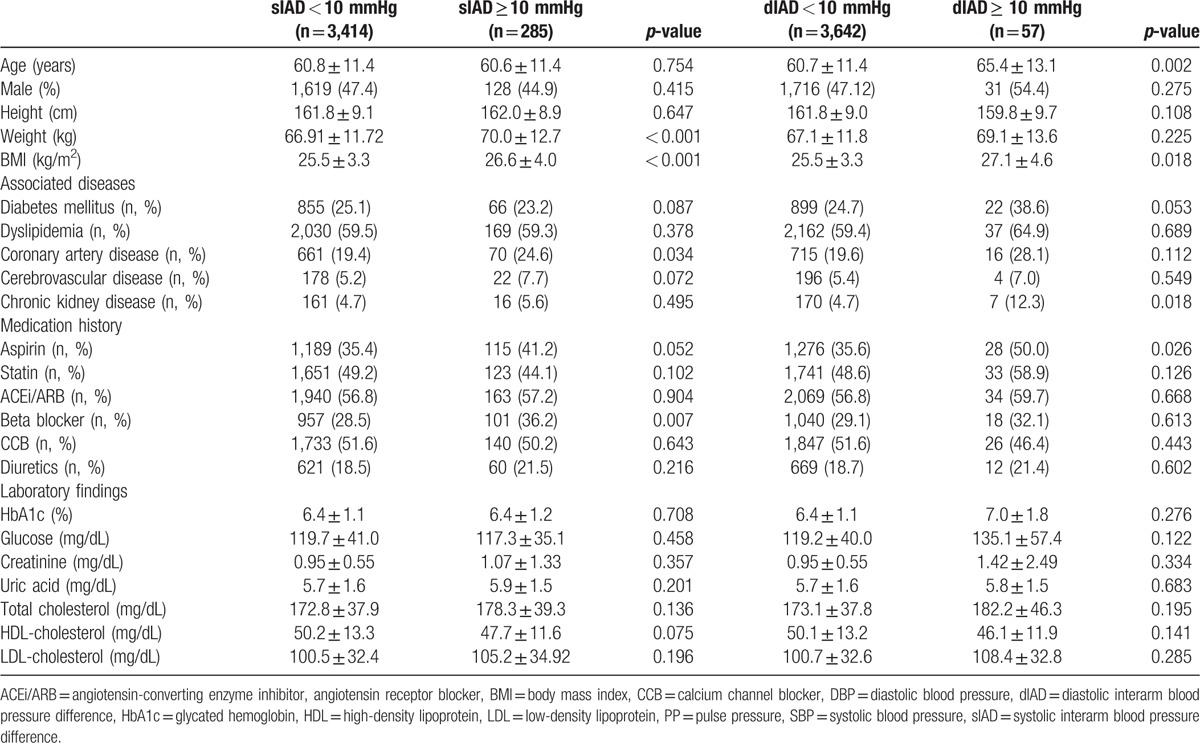
Comparison of baseline characteristics by the presence of significant interarm blood pressure difference.

**Table 5:**
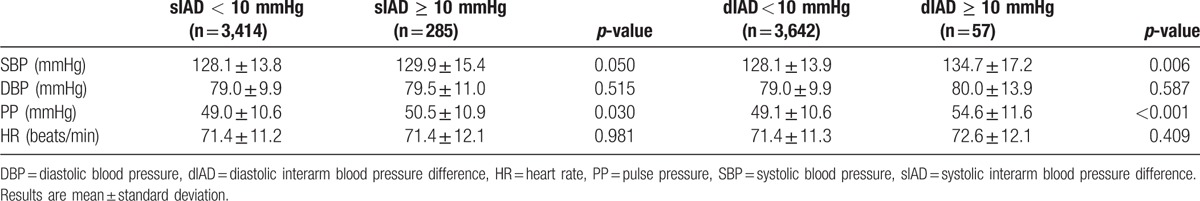
Comparison of baseline blood pressures by the presence of significant interarm blood pressure difference.

Furthermore, there were 75 patients (2.03%) with sIAD ≥ 15 mm Hg and 9 patients (0.24%) with dIAD ≥ 15 mm Hg. Patients with sIAD ≥ 15 mm Hg showed significantly higher BMI and were more likely to have diabetes (24.8% vs 32.0%, *P* = 0.006) and receive beta blockers (28.78% vs 44.59%, *P* = 0.003). Patients with dIAD ≥ 15 mm Hg were more likely to have diabetes (24.8% vs 66.7%, *P* = 0.025) (Supplement Table 1).

**Figure 2:**
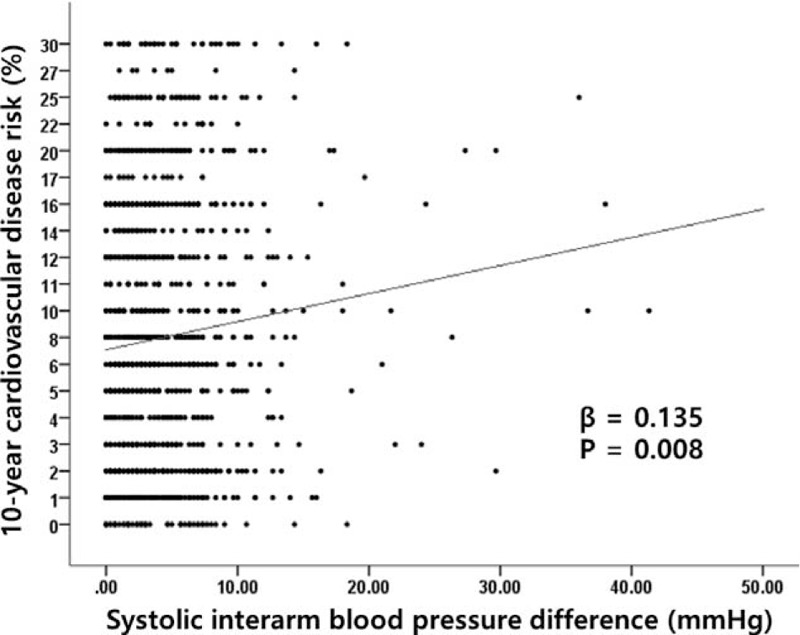
Correlation of systolic interarm blood pressure difference with the Framingham 10-y cardiovascular disease risk assessment.

The 10-year cardiovascular risk was also calculated using the Framingham risk score. The mean cardiovascular risk was 9.3 ± 7.7% in all patients, and male patients showed a higher risk of 12.9 ± 7.5% (female patients: 5.2 ± 5.5%). Results from multiple regression analysis show that the 10-year cardiovascular risk was weakly but significantly correlated with sIAD (β = 0.135, *P* = 0.008) (Fig. 2). When patients were subgrouped by a 5-mm Hg increase in sIAD (sIAD < 5 mm Hg, 5 mm Hg ≤ sIAD < 10 mm Hg, and sIAD ≥ 10 mm Hg), there were significant increases in cardiovascular risk (8.9%, 9.9%, and 10.6%, respectively, *P* = 0.037) (Table 6).

**Table 6:**
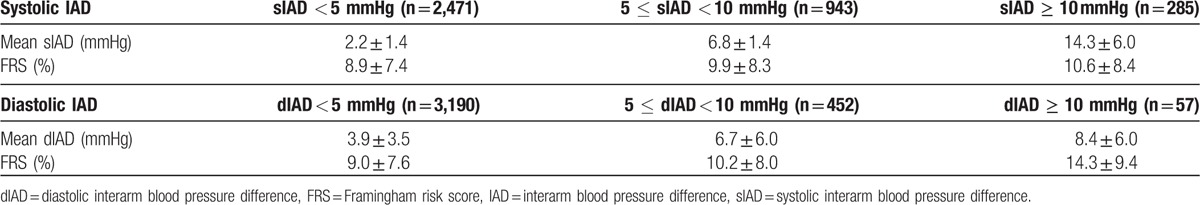
Interarm blood pressure difference and 10-year cardiovascular risk.

## 4 Discussion

The IAD is expected to be a simple, economical tool that can be used to screen patients for cardiovascular diseases. However, there are no qualified guidelines for managing these patients, and methods for BP measurement have not been standardized. Despite the fact that the Asian population has a lower prevalence of cardiovascular diseases and peripheral artery diseases than the Western population, studies on IAD with Asian samples remain scarce.^[13,14]^ There is only a consensus that the sIAD is a significant factor for atherosclerotic cardiovascular disease when the value is over 10 mm Hg. Therefore, it is important to use the IAD to determine cardiovascular risk in Asia. The standardization of methods for BP measurement should be devised through large-scale controlled studies. Utilizing 3699 participants through the establishment of a nationwide network of hypertensive patients with cardiovascular risk factors, this study shows that significant sIAD was associated with the higher prevalence of coronary artery disease and 10-year cardiovascular risk.

The present study reported a prevalence of 7.7% for significant sIAD (≥10mm Hg) in hypertensive patients with well-controlled BP. A relatively large proportion of these patients had several cardiovascular comorbidities, including diabetes mellitus, coronary artery disease, and cerebrovascular disease. However, the IAD and prevalence of patients with significant IAD in this study were lower than those in previous studies with Western populations, which reported a significant sIAD prevalence of 13% in hypertensive patients (10–19.2%).^[2]^ Another study in Japan also reported higher sIAD (4.9 ± 4.4 mm Hg; prevalence of significant sIAD, 9.1%).^[15]^ The IAD has been shown to be smaller when BP measurements are performed frequently and simultaneously.^[16]^ We measured BP 3 times simultaneously in both arms, which was more frequent than those performed in other studies. This may explain our relatively small mean IAD.

The mean SBP and DBP of patients with hypertension were 128.2 ± 13.9 and 79.1 ± 9.9 mm Hg, respectively, and 2743 (74.1%) patients showed SBP < 140 mm Hg and DBP < 90 mm Hg. More than half of all participants (1949; 52.7%) were recruited from nontertiary hospitals, which may explain why our cohort exhibited a better control rate than those in other studies. Although the mean duration of treatment for hypertension was not short (8.5 ± 11.9 years), patients with a milder form of hypertension may occupy a considerable proportion in this study. More than a half of patients were receiving combination therapy and there were small but significant differences in DBP between patients receiving monotherapy and combined therapy.

IAD does not always indicate a pathological condition. Anatomically, the left subclavian artery originates from the aorta, thus making an acute angle with the vessel. However, the right subclavian artery originates from the brachiocephalic artery without significant angulation with the originating vessel.^[17]^ The acute angle creates turbulent flow and may result in atherosclerosis,^[18]^ thereby reducing blood flow and BP.^[19]^ However, this does not explain the IAD of all patients and is rather used as a hypothesis to explain IAD in those with an unidentifiable cause.^[20]^ Instead, atherosclerotic changes in blood vessels may explain IAD more effectively. Arterial stenosis with atheromatous plaques or intimal thickening eventually causes impaired blood flow and decreased BP.^[7,21]^ Furthermore, increased vascular wall stiffness without significant stenosis is another suspicious cause of IAD.^[4,9,22]^ Recently, studies have revealed relationships between IAD and hypertension, diabetes,^[4]^ coronary heart disease,^[6]^ peripheral vascular disease,^[23]^ or other cardiovascular diseases. An association with pulse wave velocity has also been reported.^[9]^

In this study, although the mean IAD was small, the prevalence of cardiovascular disease was significantly different by sIAD. The group with significant sIAD was more likely to have coronary artery disease and cerebrovascular disease with a relative risk of 1.356 and 1.521, respectively. These findings supported the correlation between sIAD and the 10-year cardiovascular risk, as determined by the Framingham risk score. These results concur with other studies that revealed a relationship between increased sIAD and the risk or comorbidity of cardiovascular disease and cerebrovascular disease.^[2]^ However, proper consideration is needed when using the Framingham risk score with the East Asian population. It is known that the cardiovascular risk in this population is considerably lower than that of Caucasians.^[24,25]^ The Framingham risk score is also known to overestimate cardiovascular risk, especially in patients at higher risk.^[26]^ Thus the suggested 10-year cardiovascular risk in this study may not properly represent exact risk.

This study has several limitations. First, the incidence of cardiovascular disease in relation to IAD could not be predicted, and the effect of IAD control on cardiovascular events could not be evaluated. However, we plan to collect information on cardiovascular outcomes and the influence of BP control on IAD when these patients return for their 2nd visits. Second, the presence of coronary artery disease and cerebrovascular disease was only evaluated through self-completed questionnaires, and advanced studies, such as 2-dimensional echocardiography and carotid ultrasound, were selectively performed. These factors might have influenced the higher prevalence of coronary artery disease. Third, we used the Framingham risk score to evaluate the 10-year cardiovascular risk for primary prevention, although the study includes patients who have already experienced coronary artery disease. However, we also evaluated the relationship between the Framingham risk score and IAD in patients who did not have a previous history of coronary artery disease and cerebrovascular disease, and the result was not significantly different from the reported result.

The present study conducted a cross-sectional analysis in a large cohort by using an automated cuff-oscillometric device that simultaneously measured BP in both arms to determine the sIAD in hypertensive patients. Significant sIAD was correlated with both a 10-year cardiovascular risk and the presence of cardiovascular disease in well-controlled hypertensive patients. These results suggest that sIAD can be used as an additive parameter to predict future cardiovascular events in patients undergoing treatment for hypertension.

## Supplementary Material

Supplemental Digital Content
