# Significant interarm blood pressure difference predicts cardiovascular risk in hypertensive patients: CoCoNet study: Erratum

**DOI:** 10.1097/01.md.0000494761.18368.bf

**Published:** 2016-08-26

In the article “Significant interarm blood pressure difference predicts cardiovascular risk in hypertensive patients: CoCoNet study”,^1^ which appeared in Volume 95, Issue 24 of *Medicine*, several typos appeared in the original article. The article has since been corrected online.
