# Supplementary material for: Significant interarm blood pressure difference predicts cardiovascular risk in hypertensive patients: CoCoNet study
Source: Medicine (Baltimore). 2016 Jun 17;95(24):e3888. doi: 10.1097/MD.0000000000003888 (PMC4998468; doi:10.1097/MD.0000000000003888)
Supplement: Supplemental Digital Content [file medi-95-e3888-s001.doc]

Supplement Table 1. Comparison of baseline characteristics by interarm difference ≥ 15 mmHg

|  | **sIAD < 15 mmHg**  **(n = 3,624)** | **sIAD ≥ 15 mmHg**  **(n = 75)** | ***p*-value** | **dIAD < 15 mmHg**  **(n = 3,690)** | **dIAD ≥ 15 mmHg**  **(n = 9)** | ***p*-value** |
| --- | --- | --- | --- | --- | --- | --- |
| Age (years) | 60.77 ± 11.39 | 62.17 ± 11.84 | 0.292 | 60.8 ± 11.4 | 70.1 ± 13.9 | 0.014 |
| Male (%) | 1,907 (52.6) | 45 (60) | 0.205 | 1,741 (47.2) | 6 (66.7) | 0.322 |
| Height (cm) | 161.8 ± 9.0 | 161.4 ± 9.4 | 0.680 | 161.81 ± 9.0 | 156.63 ± 11.6 | 0.105 |
| Weight (kg) | 67.1 ± 11.7 | 70.2 ± 15.1 | 0.077 | 67.1 ± 11.8 | 71.3 ± 14.6 | 0.453 |
| BMI (kg/m^2^) | 25.5 ± 3.3 | 27.0 ± 5.4 | 0.025 | 25.6 ± 3.4 | 29.2 ± 6.4 | 0.149 |
| SBP (mmHg) | 128.2 ± 13.9 | 127.1 ± 16.9 | 0.558 | 128.2 ± 13.9 | 138.3 ± 26.7 | 0.290 |
| DBP (mmHg) | 79.1 ± 9.9 | 76.6 ± 13.1 | 0.105 | 79.0 ± 9.9 | 81.7 ± 21.1 | 0.722 |
| PP (mmHg) | 49.1 ± 10.6 | 50.47 ± 10.9 | 0.276 | 49.1 ± 10.6 | 56.6 ± 11.3 | 0.082 |
| HR (/min) | 71.4 ± 11.3 | 72.6 ± 13.5 | 0.428 | 71.37 ± 11.3 | 74.9 ± 11.5 | 0.387 |
| **Associated diseases** | | | | | | |
| Diabetes mellitus (n, %) | 897 (24.8) | 24 (32.0) | 0.006 | 915 (24.8) | 6 (66.7) | 0.025 |
| Dyslipidemia (n, %) | 2,157 (59.6) | 42 (56.0) | 0.739 | 2,193 (59.6) | 6 (66.7) | 1.000 |
| Coronary artery disease (n, %) | 712 (19.7) | 19 (25.3) | 0.221 | 728 (19.7) | 3 (33.3) | 0.393 |
| Cerebrovascular disease (n, %) | 194 (5.4) | 6 (8.0) | 0.297 | 199 (5.4) | 1 (11.1) | 0.394 |
| Chronic kidney disease (n, %) | 171 (4.7) | 6 (8.0) | 0.172 | 176 (4.8) | 1 (11.1) | 0.357 |
| **Medication history** | | | | | | |
| Aspirin (n, %) | 1,273 (35.7) | 31 (41.9) | 0.275 | 1,300 (35.8) | 4 (44.4) | 0.730 |
| Statin (n, %) | 1,745 (49.0) | 29 (39.2) | 0.095 | 1,768 (48.8) | 6 (66.7) | 0.332 |
| ACEi/ARB (n, %) | 2,065 (57.0) | 38 (50.7) | 0.274 | 2,096 (56.8) | 7 (77.8) | 0.315 |
| Beta blocker (n, %) | 1,025 (28.8) | 33 (45.0) | 0.003 | 1,053 (29.0) | 5 (55.6) | 0.133 |
| CCB (n, %) | 1,833 (51.5) | 40 (54.1) | 0.659 | 1,867 (51.5) | 6 (66.7) | 0.509 |
| Diuretics (n, %) | 665 (18.7) | 16 (21.6) | 0.519 | 677 (18.7) | 4 (44.4) | 0.070 |
| **Laboratory findings ­­** | | | | | | |
| HbA1c (%) | 6.37 ± 1.1.0 | 6.5 ± 1.1 | 0.737 | 6.4 ± 1.1 | 6.7 ± 0.7 | 0.643 |
| Glucose (mg/dL) | 119.5 ± 40.6 | 121.1 ± 37.5 | 0.810 | 119.5 ± 40.6 | 120.8 ± 21.1 | 0.937 |
| Creatinine (mg/dL) | 1.0 ± 0.5 | 1.5 ± 2.4 | 0.230 | 1.0 ± 0.7 | 0.9 ± 0.3 | 0.930 |
| Uric acid (mg/dL) | 5.7 ± 1.6 | 5.7 ± 1.4 | 0.856 | 5.7 ± 1.6 | 6.14 ± 1.7 | 0.522 |
| Total cholesterol (mg/dL) | 173.1 ± 38.0 | 179.9 ± 37.0 | 0.303 | 173.2 ± 38.0 | 182 ± 38.5 | 0.573 |
| HDL-cholesterol (mg/dL) | 50.1 ± 13.2 | 46.5 ± 11.9 | 0.146 | 50.0 ± 13.2 | 43.4 ± 6.8 | 0.261 |
| LDL-cholesterol (mg/dL) | 100.8 ± 32.5 | 106.7 ± 37.5 | 0.376 | 100.8 ± 32.6 | 119.2 ± 36.0 | 0.208 |

ACEi/ARB, angiotensin-converting enzyme inhibitor, angiotensin receptor blocker; BMI, body mass index; CCB, calcium channel blocker; DBP, diastolic blood pressure; dIAD, diastolic interarm blood pressure difference; HDL, high-density lipoprotein; HR, heart rate; IAD, interarm blood pressure difference; LDL, low-density lipoprotein; PP, pulse pressure; SBP, systolic blood pressure; sIAD, systolic interarm blood pressure difference.

Unless otherwise stated, results are mean ± standard deviation.
